# Effectiveness of interceptive treatment of class III malocclusions with skeletal anchorage: A systematic review and meta-analysis

**DOI:** 10.1371/journal.pone.0173875

**Published:** 2017-03-22

**Authors:** Jorge Rodríguez de Guzmán-Barrera, Carla Sáez Martínez, Montserrat Boronat-Catalá, Jose María Montiel-Company, Vanessa Paredes-Gallardo, José Luís Gandía-Franco, José Manuel Almerich-Silla, Carlos Bellot-Arcís

**Affiliations:** 1 Department of Stomatology, Faculty of Medicine and Dentistry, University of Valencia, Valencia, Spain; 2 Preventive Dentistry Teaching Unit, Department of Stomatology, Faculty of Medicine and Dentistry, University of Valencia, Valencia, Spain; 3 Orthodontics Teaching Unit, Department of Stomatology, Faculty of Medicine and Dentistry, University of Valencia, Valencia, Spain; Virginia Commonwealth University, UNITED STATES

## Abstract

Recently, new strategies for treating class III malocclusions have appeared. Skeletal anchorage appears to reduce the dentoalveolar effects while maximising the orthopaedic effect in growing patients. The purpose of this systematic review and meta-analysis is to examine the effectiveness of bone anchorage devices for interceptive treatment of skeletal class III malocclusions. Searches were made in the Pubmed, Embase, Scopus and Cochrane databases, as well as in a grey literature database, and were complemented by hand-searching. The criteria for eligibility were: patients who had undergone orthodontic treatment with skeletal anchorage (miniplates and miniscrews). Patients with syndromes or craniofacial deformities or who had undergone maxillofacial surgery were excluded. The following variables were recorded for each article: author, year of publication, type of study, sample size, dropouts, demographic variables, treatment carried out, radiographic study (2D or 3D), follow-up time, and quality of the articles on the Newcastle-Ottawa Scale. The means and confidence intervals of the following variables were employed: Wits, overjet, ANB, SNA and SNB. Initially, 239 articles were identified. After removing the duplicates and applying the selection criteria, 9 were included in the qualitative synthesis and 7 in the quantitative synthesis (meta-analysis). It may be concluded that skeletal anchorage is an effective treatment for improving skeletal Class III malocclusion, but when compared with other traditional treatments such as disjunction and face mask, there is no clear evidence that skeletal anchorage improves the results.

## Introduction

The incidence of skeletal class III malocclusion varies in different population types. It is around 5% in patients of Caucasian origin but between 9% and 19% in those of Asian descent [1]. It can be caused by a retrognathic or hypoplastic maxilla, a prognathic mandible, or a combination of the two [2,3].

The patient’s age and growth stage are decisive factors in treating this craniofacial disharmony. In adults, the treatment can be a combination of orthodontics and surgery, or just orthodontic camouflage. At a young age, orthopaedic treatment aims to reduce future therapeutic needs in the permanent dentition [4]. Traditionally, a face mask, chin cup or functional appliance has been employed [5–7]. In recent years, however, a number of authors have carried out orthopaedic treatment using skeletal anchorage [8–10]. It would appear that using miniplates and miniscrews achieves greater skeletal effects with a smaller dentoalveolar component than is the case with conventional orthopaedic appliances [11–13].

However, skeletal anchorage presents certain drawbacks: these are invasive treatments that require surgery both to insert and remove them, and some of the components are not stable throughout the treatment [14]. Postoperative inflammation, irritation of adjacent tissues in contact with the mini screws and buildups of food scraps in the area are reported as side effects [15]. Nevertheless, the use of skeletal anchorage for orthopaedic treatment of skeletal class III malocclusion continues to spread. Consequently, the purpose of this systematic review and meta-analysis is to examine the effectiveness of interceptive treatment of class III malocclusions using skeletal anchorage.

## Materials and methods

The bibliography was reviewed systematically, following the PRISMA (Preferred Reporting Items for Systematic Reviews and Meta-Analyses) recommendations [16]. The systematic review was registered with the PRISMA (PROSPERO) database, reference number CRD42015027846.

### Selection criteria for the studies included in this review

The criteria for inclusion were: articles, articles in press and reviews concerning studies in humans. Only systematic reviews, meta-analyses, randomized clinical trials (RCTs), case-control studies and cohort studies were accepted. Both retrospective and prospective studies were included. Case reports, case series, literature reviews, systematic reviews, meta-analyses and editorials were excluded.

The criteria for eligibility were: studies in growing patients with skeletal class III malocclusion who had undergone orthodontic treatment with skeletal anchorage, including miniplates and miniscrews Patients with syndromes or craniofacial deformities or who had undergone maxillofacial surgery were excluded.

### Search strategy

To identify relevant studies irrespective of language, a rigorous electronic search was made in the Pubmed, Scopus, Cochrane Library and Embase databases. An electronic search for “grey literature” was also made in the New York Academy of Medicine Grey Literature Report. The search was made with no time limit and was updated in September 2016.

A list of both Mesh and non-Mesh terms was drawn up to confine the research field to articles that were directly related to the study subject. The search string included a series of terms referring to skeletal anchorage (“(skeletal anchorage OR bone anchor OR miniscrew OR miniplate OR mini implant OR bone screw OR bone plate)”) combined with terms related to skeletal class III malocclusion (“(skeletal class III OR mandibular prognathism OR mandibular hyperplasia OR maxillary retrusion OR maxillary hypoplasia OR mandibular protrusion OR angle class III)”) and orthopaedic treatment (“(interceptive treatment OR early treatment OR orthopedic treatment OR interceptive orthodontic OR interceptive OR early therapy OR children)”). Different combinations of these terms were considered and the search was completed by hand searching for the bibliographical references cited in the articles included, in order to add studies that had not been found during the primary search.

Two reviewers (M.B-C and C.S-M) independently assessed the titles and abstracts of all the articles identified. The Kappa score [17] was used to measure the degree of agreement about selection on reading the title and abstract. In the event of any disagreement, a third reviewer (J.R.G-B) was consulted. When the abstract did not contain sufficient information to reach a decision, the reviewers read the full article before taking the final decision. The full texts of all the articles were then read and the reasons for rejecting those excluded were recorded.

### Data mining and list of variables

The following variables were recorded for each article reviewed: author, year published, type of study (retrospective, prospective, controlled, not controlled), sample size, dropouts, demographic variables (gender, age), inclusion and exclusion criteria, treatment used, type of radiographic study used (2D or 3D), follow-up time, quantitative or qualitative variables expressing the study results, and quality of the articles accepted.

### Quality assessment

The quality of the studies was analysed by the same researchers, independently, using the Newcastle-Ottawa Scale [18]. In the event of discrepancy between the initial two researchers a consensus was reached and in case of doubt, the third researcher was consulted.

### Measurement of the variables and synthesis of the results

The means and confidence intervals of the following variables were calculated: Wits, overjet, ANB, SNA, SNB.

### Statistical analysis

For the quantitative synthesis, the inter-group differences in means and their confidence intervals were determined for all the studies included in this meta-analysis. Heterogeneity was assessed with the Q test and the I^2^ test. The DerSimonian-Laird random effects pooling method was used to calculate the differences in weighted means. Rosenthal's fail safe number was used to assess publication bias.

## Results

### Study selection and flow diagram

**Fig 1:**
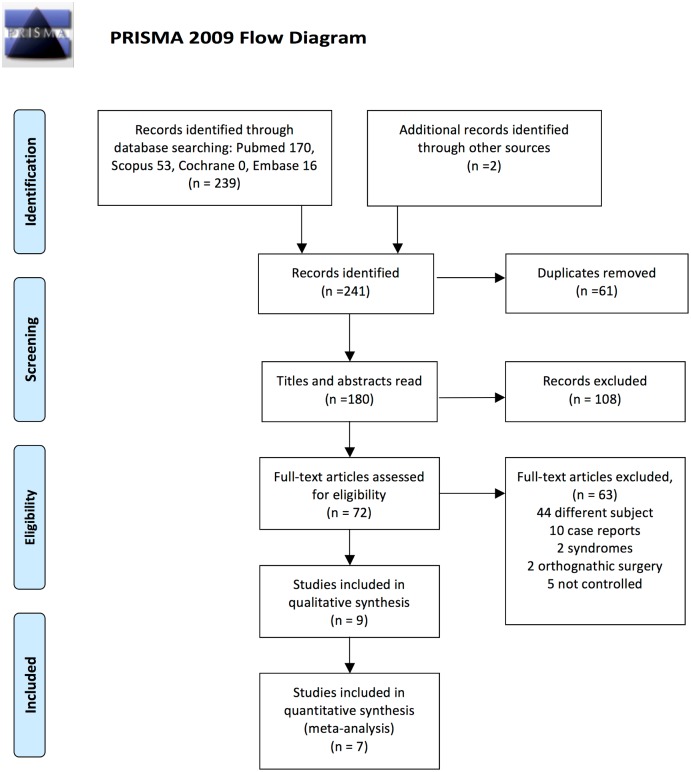
The PRISMA flow diagram. *From*: Moher D, Liberati A, Tetzlaff J, Altman DG, The PRISMA Group (2009). *P*referred *R*eporting *I*tems for *S*ystematic Reviews and *M*eta*-A*nalyses: The PRISMA Statement. PLoS Med 6(7): e1000097. doi:10.1371/journal.pmed1000097. **For more information, visit** www.prisma-statement.org.

A thorough search identified 239 articles in total: 170 in Pubmed, 53 in Scopus, none in the Cochrane Library and 16 in Embase. A further 2 references that had not been found through the primary search were added manually. After removing 61 duplicates, 107 were excluded on reading the title and abstract as they were unrelated to the research question. The Kappa score for inter-assessor agreement was 0.89. The remaining 73 articles were read and analysed. On reading their full texts, 63 were excluded for the following reasons: 44 were unrelated to the research question, 10 were case series, 4 did not meet the inclusion criteria and 5 had no control group. Finally, 9 articles met the inclusion criteria and were included in the qualitative synthesis and 7 were included in the quantitative synthesis (meta-analysis). The PRISMA flow chart (Fig 1) gives an overview of the article selection process.

### Study characteristics

The 9 studies included presented moderate quality on the Newcastle-Ottawa Scale [18] (Table 1).

**Table 1:** Quality of the studies on the Newcastle-Ottawa Scale.

Author/year [reference]	SELECTION (****)				COMPARABILITY (**)	EXPOSURE (***)		
	Case definition adequate	Representativeness of cases	Selection of controls	Definition of controls	Comparability of cases & controls	Ascertainment of exposure	Same method of ascertainment for cases & controls	Non-response rate
şar et al. (2014) [19]	*	*	*	*	*		*	
Niemkemper et al. (2015) [20]	*	*	*	*	*		*	
Hino et al. (2013) [22]	*	*	*	*	*		*	
Ge et al. (2012) [23]	*	*	*	*	*		*	
Cha & Ngan (2011) [24]	*		*	*	*		*	
Baccetti et al. (2011) [21]	*	*	*	*	*		*	
De Clerck & Swennen (2010) [14]	*	*	*	*	*		*	
Cevidanes et al. (2010) [25]	*	*	*	*	*		*	
Koh & Chung (2014) [26]	*	*	*	*	*		*	

* = 1 point.

(**) = up to 2 points.

(***) = up to 3 points.

(****) = up to 4 points.

Of the 9 studies, 8 were case-control studies and 1 was a controlled clinical study. In all 9, a group of patients treated with miniplates, miniscrews or any other type of bone anchorage device was compared with a control group. In 4 of the 9 studies the control group did not receive any type of treatment [14,19–21]. In the other 5, the control group was treated with a rapid maxillary expander and a face mask [22–26].

The patients in the studies selected had Class III skeletal and molar malocclusion, a Wits appraisal of ≤ -1mm, anterior crossbite and/or edge to edge occlusion, and some type of indication that the patient was still growing, such as mixed dentition or cervical vertebral maturation stage CS1-CS3.

### Qualitative synthesis of the studies included

In 2 of the studies the skeletal anchorage device consisted of microscrews [20–23], while the remaining 7 used miniplates [14,19,21,22,24–26]. As regards the location of the miniplates and miniscrews in the craniofacial structures, Şar et al. [19] placed miniplates at the level of the maxillary part of the lateral nasal wall and in the symphysis mentalis. Cha & Ngan [24] placed them at the level of the zygomatic process, while other authors [14,21,22,25] situated them in this area and in the mandible between the lateral incisors and the lower canines. For their part, Niemkemper et al. [20] positioned miniscrews on both sides of the mid-palatal suture and Ge et al. [23] set them in the zygomatic arch. Lastly, Koh & Chung [26] did not specify the position of the miniplates on the maxilla. Table 2 presents the selected studies, showing the sample size, dropouts, demographic variables, inclusion and exclusion criteria, treatment used, type of radiographic study (2-D or 3-D), follow-up time, variables studied, quality of the articles and conclusions.

**Table 2:** Summary of the studies included in the qualitative analysis.

Author (year) [reference] Type of study	N (dropouts) Ca(cases) Co(controls) %M(n), %W(n) Mean age	Inclusion criteria (In) Exclusion criteria (Ex)	Ca (case group appliances) Co (control group appliances)	Measurements (2D/3D)	Follow-up time	Conclusions	Quality (Newcastle-Ottawa Scale)
şar et al. (2014) [19] Case-Control	51(-) Ca (34) Co (17) -%M(-) -%W(-) Ca (G1: 11.23±1.48. G2: 11.25±1.52) Co (9.87±1.20)	In: 1. Dental & skeletal CIII with maxillary deficiency, 2. Meso/brachy, 3. ACB and molar CIII, 4. Overbite normal/ increased. Ex: -	Ca (G1 FM to MPs on anterior maxillary wall, G2 intermaxillary CIII elastics from MPs at symphysis to appliance cemented to maxilla) Co (no treatment)	**Facial height**: ANS-Me, N-Me. **ntermaxillary skeletal variables**: ANB, Wits, A-vertical plane, B-vertical plane. **Max inc**: V1-pp. **Max molar**: U6-VRmx. **Mand Inc**: L1MP. **Soft tissue profile**: A’-VR, UL-VR, LL-VR, Pg’-VR (2D)	**MP+FM**: 7.4m **MP+CIII Elastics**: 7.6 m **Control Group**: 7.5m	Miniplates with a face mask or Class III elastics are a good alternative to conventional methods in severe skeletal Class III malocclusion cases. Miniplates with a face mask are preferable in patients with severe maxillary retrusion and a vertical pattern, while miniplates with elastics are preferable in patients with a normal or brachyfacial pattern.	6/8
Niemkemper et al. (2015) [20] Case-Control	32(-) Ca (16) Co (16) 56.3%M(18) 43.7%W(14) Ca (9.5±1.6) Co (9.4±1.1)	In: 1. Molar and skeletal CIII, mixed dentition, 2. Wits ≤-2, 3. ACB or edge to edge Ex: -	Ca (hybrid Hyrax + face mask) Co (no treatment)	**Cranial Base**: N-S-Ba. **Maxillary**: SNA, PtA-NaPerp, Co-Pt A. **Mandibular**: SNB, Pog-NaPerp, Co-Gn. **Maxillary-mandibular**: Wits, ANB. **Vertical**: FMA, Co-Go-Me. **Interdental**: overjet, overbite, molar relationship, interincisal angle. **Dentoalveolar**: U1-FH, U1-PP, L1-MP (2D)	**Treated Group**: 0.9±0.4y **Control Group**: 1.0±0.5y	The Hyrax FM combination is a very effective treatment in growing Class III patients. It achieves significant maxillary advancement and improved mandibular sagittal position. There is less need for invasive surgical treatment than with skeletal anchorage devices.	6/8
Hino et al. (2013) [22] Case-Control	46 (-) Ca (25) Co (21) 37%M (17) 63%W (29) Ca (11.9±1.8) Co (8.1±1.5)	In: 1. Skeletal CIII, 2. Wits ≤-1, 3. ACB/edge to edge, 4. Molar CIII or mesial step, 5. Stages 1–3 of cervical vertebral maturation Ex: -	Ca (Bone anchored maxillary protraction- BAMP) Co (Face mask+RME)	Colour maps on overlaid T1 & T2 CBCT images: maxilla, upper incisor, right zygoma, left zygoma. (3D)	**BAMP group**: 1.2±1y **RME/FM group**: 10.1± 2.2m	Orthopaedic changes can be achieved with both the RME/FM and BAMP protocols. Approximately half of the patients treated with RME/FM underwent greater dental than skeletal changes, and in a third of those treated with RME/FM and a sixth of those treated with BAMP the displacement was mostly vertical.	6/8
Ge et al. (2012) [23] Case-control	49 (6) Ca (25) Co (24) 47%M (23) 53%W (26) Ca (10y4m) Co (10y6m)	In: 1. Prepubertal CVM stage, 2. Dental & skeletal CIII, maxillary deficiency, 3. ANB<0, 4. Wits ≤-2, 5. ACB, 6. Overbite. Ex: systemic illnesses or congenital deformities.	Ca (Facemask in association with miniscrew implants—MSI/FM) Co (Facemask with RME—RME/FM)	**Maxillary**: SNA, Co-A, A-Nperp. **Mandibular**: SNB, Co-Gn, Pog-Nperp. **Maxillomandibular**: ANB, Wits, A-N-Pog. **Dental**: U1-SN, U1-NA, IMPA, L1-NB, Overbite, Overjet, U1-VR, U1-HR, U6-VR, U6-HR. **Vertical**: PP-SN, SN-MP, N-GN, S-Go. **Soft tissue**: UL-E plane, LL-E plane, ST convexity (2D)	**MSI/FM group**: 11m **RME/FM group**: 1y1m	Miniscrews can be used as reliable means of rigid anchorage for maxillary protraction. The zygomatic crest of the maxilla is an important region for skeletal anchorage placement. In comparison with RME/FM, MSI/FM produces similar maxillary advancement and a mandibular restriction in CIII patients with maxillary deficiency while using a lower protraction force. The MSI/FM protocol improves bone and soft tissue relationships. It also eliminates the unwanted tooth movement that occurs with RME/FM treatment.	6/8
Cha & Ngan (2011) [24] Case-Control	50 (-) Ca (25) Co (25) 38%M (19) 62%W (31) Ca (11±1.4) Co (10.8±0.9)	- Not stated	Ca: Face mask with miniplate anchorage (FM+MP) Co: RME+face mask (FM+RME)	SNA, SNB, ANB, A-N perpendicular to FH, N-A-Pog, SON, Co-A, Co-Pog, S-N, S-Ba, SN-Ba, SN-SBa, PP-FH, FMA, ANS-Me, Mx1-FH, IMPA, nasolabial angle, UL-E line, LL-E line (2D)	**FM+MP group: 9.9**±2.4m **FM+RME group**: 8.5±2.4 m	The maxillary advancement was greater in the group treated with miniplates than in the RME group. Mesial movement of the teeth was not observed in the miniplate group, unlike the RME group. Extrusion of the upper first molar was greater in the RME group, increasing the lower face height.	6/8
Baccetti et al. (2011) [21] Case-Control	41 (-) Ca (26) Co (15) 48.8% H (20) 51.2%W (21) Ca (11.9±1.8) Co (9.6±1.6)	In: 1. CIII mixed/ permanent teeth, 2. Witts ≤-1, 3. ACB/edge to edge, 4. Molar Class III, 5. Caucasian, 6. prepubertal CVM stage. Ex: -	Ca: Bone anchored maxillary protraction (BAMP) Co: No treatment	Comparison of points at T1 and T2 using Viewbox software: A, B, Pr, Id, Gn, Me, TgGo1, Go, TgGo2, Ar, Co, Cs, Ptm, Ba, ANS, PNS. (3D)	**BAMP group**: 1.2±1.0 y **Control group**: 1.6±1.0 y	In the BAMP group the maxilla was extended horizontally in an anterior direction (at PNS and PTM level) and the mandible was deformed horizontally in a posterior direction. However, in the control group an upwards and backwards deformation of the condyle appeared, as did deformation of the horizontal plane in an anterior direction at symphysis mentalis level and in a posterior direction in the maxilla.	6/8
De Cleck y Swennen (2011) [14] Case-Control	39 (-) Ca (21) Co (18) -%M(-) -%W(-) Ca (11.10±1.8) Co (11.6±1.7)	In: 1. CIII malocclusion in primary dentition, 2. Wits ≤-1mm, 3. ACB or edge to edge, 4. Molar CIII, 5. CVM Stages 1–3. Ex: -	Ca: 4 miniplates and intermaxillary elastics (BAMP Protocol). Co: not treated.	**Base of the cranium**: Or, Pg, A, Co, Ptm, Or, Pr, Id, B, Pg to VertT. Co-Gn, Co-Go, Go-GN, Wits. **Maxillomandibular**: ANB, Wits. **Soft Tissue**: ANS, A, Ulip, Llip, B, Pg to VertT. **Dental**: U1-NL, L1-ML. **Interdental**: Overjet, Overbite, Molrel. **(3D)**	**BAMP group**: 1y **Control group**: 1y	The BAMP protocol induces greater maxillary advancement than RME/FM. The sagittal mandibular changes were similar, while the vertical changes were controlled better with BAMP.	6/8
Cevidanes et al. (2010) [25] Case-Control	55(-) Ca (34) Co (21) 43%M(24) 56%W(31) Ca (8.3±1.47)(-10) Co (11.10±1.10)	In: 1. skeletal and dental CIII in primary dentition, 2. Wits ≤ -1mm, ACB or edge to edge, 3. Caucasian ethnic group, 4. CVM Stages CS1-CS3. Ex: -	Ca: 4 miniplates and intermaxillary elastics (BAMP Protocol). Co: RME+face mask (FM+RME)	A, Co, B, Pg to VertT. Co-Gn, Co-Go, Go-Gn. **Maxillomandibular**: ANB, Wits. **Dental**: U1-NL, L1-ML. ANS-Me, Co-Go-Me. **Interdental**: Overjet, Overbite, Molrel. **(3D)**	**BAMP group**: 1y. **RME+FM**: 10m	The BAMP protocol induces greater maxillary advancement than RME/FM. The sagittal mandibular changes were similar, while the vertical changes were controlled better with BAMP.	6/8
Koh & Chung (2014) [26] Controlled clinical study	47(-) TBFM(28) SAFM (19) TBFM 7M/21W (9–13.9) 10.09 SAFM 8M/11W (9.1–13.0) 11.21	In: 1. overjet >-2 mm, 2. No craniofacial deformity, 3. Stages CS3-CS4, 4. No prior orthodontic or surgical treatment. Ex: -	**TBFM**: FM+Hyrax (64+46) bands. 1–2 activations/day. 1. FMH: tooth-borne FM/High angle type. 2. FML: tooth-borne FM/low agle type. 3. FM3: tooth/borne FM/CVM3. 4. FM4: tooth-borne FM/CVM4. **SAFM**: MP with 4 MS, FM + 400-500gr elastics. 1. SAH: skeletal anchorage FM/High angle type. 2. SAL: skeletal anchorage FM/Low angle type. 3. SA3: skeletal anchorage FM/CVM3. 4. SA4: skeletal anchorage FM/CVM4	SNA, SNOr, Mx Length, N-A, Palatal P, SNB, Mn. Length, N-Pog. **Maxillomandibular**: ANB, A-B to Mn P, FMA. **Dental**: U1-SN, IMPA.	-	SAFM led to a large increase in all anterior-posterior measurements. The changes that took place in the two high vertical type groups show that the SAFM group presented greater anterior movement of the orbitale and a reduction in the mandibular plane. Within the same cervical vertebra maturation stage, at CVM3 the anterior-posterior movement was greater with SAFM than with TBFM.	6/8

Abbreviations: M: men, W: women, y: years, m: months; CIII: class III malocclusion, Meso/braqui: mesofacial/brachyfacial, ACB: anterior crossbite, CVM: cervical vertebral maturation; FM: face mask, MP: miniplates, MS: miniscreews, RME: rapid maxillary expansion appliance; Max inc: maxillary incisor, Max molar: maxillary molar, Mand inc: mandibular incisor.

To assess the treatment employed, Hino et al. [22] superimposed CBCT images at T1 and T2 through a colour map and Bacceti et al. [21] compared points at T1 and T2 by means of a thin-plate spline using Viewbox software. The other 7 studies [14,19,20,23–26] employed similar parameters. To examine the antero-posterior relationship of the maxillae, all these authors used ANB and Wits appraisal and some also used SNA and SNB [20,23,24,26]. Additionally, 4 of the 7 articles also recorded overjet and overbite [14,20,23,25].

### Quantitative synthesis of the studies included

**Fig 2:**
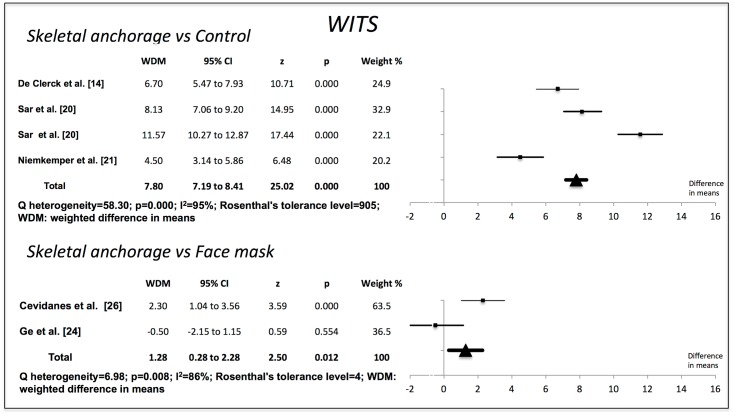
Changes in Wits (mm). Effectiveness of skeletal anchorage compared to control group and to expander and face mask.

**Fig 3:**
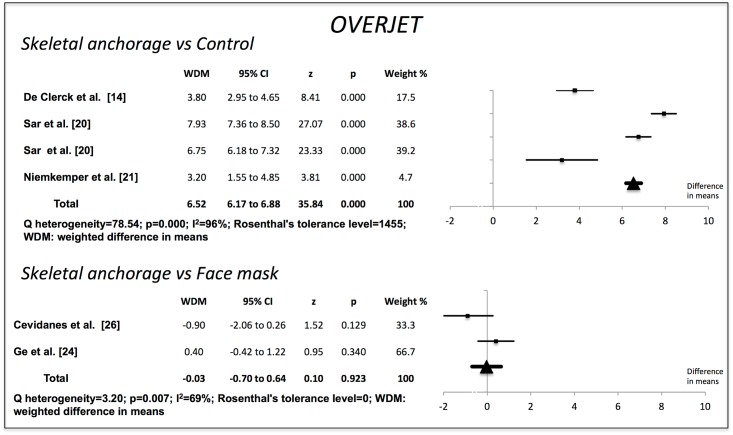
Changes in overjet (mm). Effectiveness of skeletal anchorage in comparison with the control group and with the expander and face mask group.

**Fig 4:**
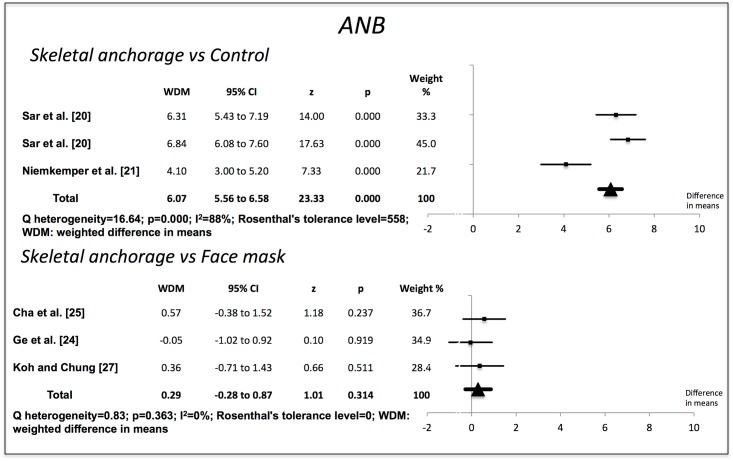
Changes in ANB (degrees). Effectiveness of skeletal anchorage compared to control group and to expander and face mask.

**Fig 5:**
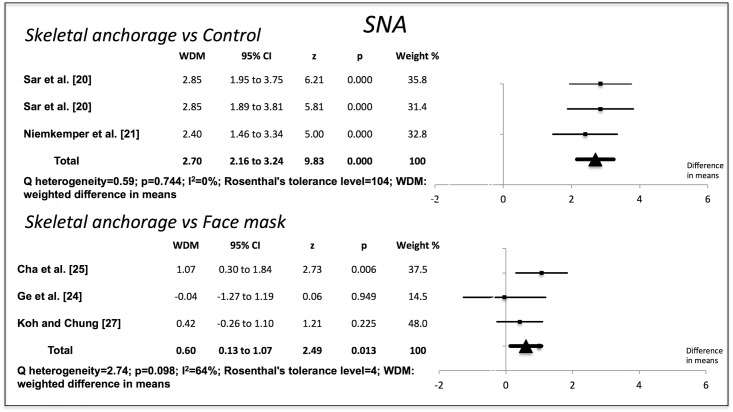
Changes in SNA (degrees). Effectiveness of skeletal anchorage compared to control group and to expander and face mask.

**Fig 6:**
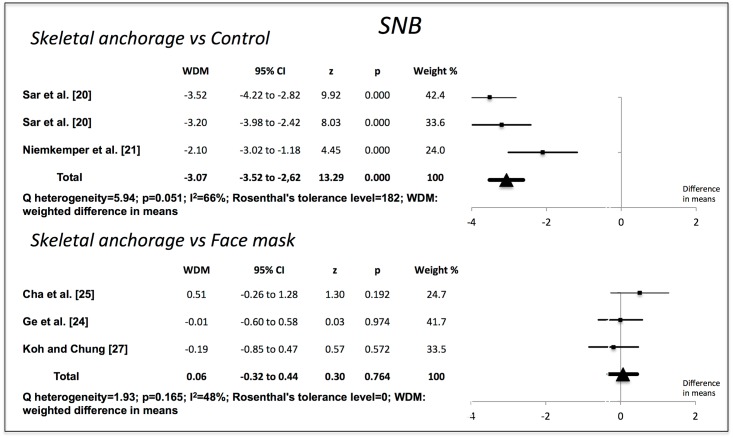
Changes in SNB (degrees). Effectiveness of skeletal anchorage compared to control group and to expander and face mask.

Compared to the no-treatment control groups, the skeletal anchorage groups showed significant changes in all the variables examined. The Wits value increased by 7.80 mm (95% CI 7.19–8.41) (p = 0.000). Overjet increased by 6.52 mm (95% CI 6.17–6.88) (p = 0.000). ANB increased by 6.07° (95% CI 5.56–6.58) (p = 0.000). The SNA increased by 2.70° (95% CI 2.16–3.24) (p = 0.000). SNB decreased by 3.07° (95% CI -3.52 to -2.62) (p = 0.000). The studies used to obtain the weighted mean differences for Wits, overjet and ANB showed a high degree of heterogeneity (I^2^>85%), but those used for SNA and SNB were more homogenous (Figs 2–6).

On comparing the skeletal anchorage treatments with the expander and face mask groups, the difference in mean Wits increased by a significant 1.28 mm (95% CI 0.28–2.28) (p = 0.012). Overjet did not present significant differences, however (-0,03 mm; 95% CI -0,70 to 0,64) (p = 0.923), nor did ANB (0.29°, 95% CI -0.28 to 0.87) (p = 0.314). SNA, on the other hand, did (0.60°, 95% CI 0.13–1.07) (p = 0.013), although SNB did not (0.06°, 95% CI -0.32 to 0.44) (p = 0.764). The studies used in the Wits appraisal comparison exhibited high heterogeneity (I^2^ = 86%). The heterogeneity in overjet was I^2^ = 69%. For ANB, SNA and SNB it was I^2^ = 0%, I^2^ = 64% and I^2^ = 48% respectively.

### Publication bias

Rosenthal’s tolerance level was high for the meta-analysis of studies comparing skeletal anchorage with a control group, indicating a very low publication bias impact. For the comparisons between skeletal anchorage and the use of an expander and face mask, however, Rosenthal’s tolerance level was very low.

## Discussion

The use of skeletal anchorage in orthodontic treatments has been gaining popularity among orthodontists. Since the first publications on the subject, the use of these methods has increased. Nevertheless, most of the published work has consisted of clinical case or case series reports illustrating new treatment approaches. However, there is no consensus regarding indications, techniques, protocols, age for treatment, treatment times, the forces employed or the results obtained.

The present systematic review, conducted in accordance with the PRISMA criteria [16] (S1 Table) shows the lack of randomised controlled trials. Additionally, the heterogeneity of the studies does not provide the best conditions for meta-analysis. It should not be forgotten that the 9 articles included are the result of a very exacting selection process. Many articles published as clinical cases or case series have not been included in the meta-analysis but have provided very valuable information for subsequent studies of higher evidence quality [27].

Most of the authors have encountered more advantages than drawbacks in using skeletal anchorage with miniplates and miniscrews in the orthopaedic treatment of skeletal Class III malocclusions (Table 3). It should be noted that because miniplates are intraoral, they are more comfortable than facemasks and make it possible to keep the elastic bands in place 24 hours a day.

**Table 3:** Advantages and disadvantages of skeletal anchorage according to the studies reviewed.

ADVANTAGES	DISADVANTAGES
Fewer unwanted dental effects than with dental anchorage [14,19–24] Greater maxillary advancement [14,19–21,24,25] Greater skeletal effect [19,22,23] The force vector passes through the centre of resistance of the maxilla [19] Less anti-clockwise [US: counterclockwise] rotation of the maxilla [19] Intermaxillary elastic bands with skeletal anchorage can be worn 24h a day [19] Greater facial profile improvement [19] Intermaxillary elastics with skeletal anchorage do not require as much cooperation as with dental anchorage [19] Vertical changes do not appear in any of the craniofacial structures [21] Greater improvement in overjet and molar relation [14] Lower traction force needed with elastics (up to 250 g per side) [22,23] Possible to achieve maxillary advancement in older patients than with dental anchorage [24] Less clockwise rotation of the mandible [24] Dental alignment can be performed simultaneously [24]<0}	Invasive surgical procedure [20] Two surgical procedures: placement and removal of miniplates [20,22] More expensive [19] Requires general anaesthetic or sedation [19] Possibility of failure of the skeletal anchorage (miniplates or miniscrews) [23,27]

Traditionally, the recommendation has been to start maxillary traction with a face mask during the early mixed dentition stage (around 8 years old) in order to achieve maximum skeletal effect, as the sutures’ capacity to respond to treatment diminishes with age. With skeletal anchorage, however, the age for starting treatment is later, around 10 years old, when the characteristics of the bone facilitate its placement and favour its stability [14,19,22,24].

According to the studies included in this review, candidates for treatment with skeletal anchorage devices will present skeletal Class III malocclusions in a growing patient with maxillary deficiency, dental Class III malocclusion, Wits ≤-1, anterior crossbite or edge-to-edge occlusion and stage CS1-CS3 cervical vertebra maturation [14,20–23,25].

Although skeletal anchorage is an invasive surgical procedure [20], requiring two separate operations to insert and remove the miniplates [20,22], none of the studies assessed the quality of life of the patients treated by this method.

There is some variation in the treatments used for these malocclusions. According to the studies, the BAMP (Bone Anchored Maxillary Protraction) protocol consisted in attaching 4 miniplates: 2 on the infrazygomatic crest of the maxilla and 2 between the lower lateral incisors and canines. The initial force was 100 g on each side, gradually rising to 250 g per side [8,21,22]. In the Hybrid Hyrax Facemask combination, a hybrid Hyrax was combined with 2 miniscrews at the palatine suture and 2 elastic bands on the first molars. The force applied was 400 g on each side [20]. In the MSI/FM (Miniscrew Implants/ Face Mask) protocol, 2 miniscrews were placed on the zygomatic arch and loading began 2 weeks later, using elastic bands to apply a force of 200/250 g on each side for 14 hours a day [23,24] used miniplates fixed to the zygomatic arch with 3 miniscrews, applying a force of 400 g on each side after an interval of 3–4 weeks. In one group, Şar et al. [19] placed 2 miniplates on the maxillary section of the lateral nasal wall and 7 days later applied a force of 400 g on each side for 16 hours a day using a face mask. In the other group, they placed 2 miniplates on the symphysis with intermaxillary elastic bands to a maxillary expander cemented to the upper arch. A week later they applied a force of 500 g for 24 hours a day. Lastly, Koh & Chung [26] used the SAFM (Skeletal Anchorage Facemask) protocol, consisting in attaching miniplates and applying an elastic force of around 400–500 g per side. Loading began 2–3 weeks after surgery. The patients had to wear the face mask 24 hours a day, except during meals.

As regards treatment stability, Ge et al. [23] concluded that maxillary protraction treatment with skeletal anchorage reduces the possibility of relapse because most of the advancement is strictly skeletal and there is no unwanted dental movement.

Radiographic diagnosis was performed in 3D using CBCT in 4 studies [14,21,22,25] and in 2D in the rest, using lateral cranial teleradiography before and after treatment [19,20,23,24,26].

Of the 9 studies that investigated the effects of Class III treatment with skeletal anchorage, Hino et al. [22] used a system based on a colour map to analyse the changes and size of the changes achieved by the treatment, while Baccetti et al. [21] used a morphometric analysis to study the variations in the shape of the dentofacial structures. Owing to the methods they employed, these two studies are not comparable with the others.

In the other 7 studies there was considerable variation in the measurements used to measure the effects of bone anchorage on the orthopaedic treatment of skeletal Class III malocclusion. Several of the studies used SNA, SNB, ANB, U1-PP, L1-MP, Wits appraisal, overjet and overbite [19,20]. Ge et al.[23] used the same variables except for U1-PP. Cha & Ngan [24] used the same variables with the exception of overjet and overbite as well as U1-PP. Of this group of variables, Koh & Chung [26] only employed SNA, SNB and ANB. Lastly, De Clerck & Swennen [14] and Cevidanes et al. [25] used similar methods and the same measurements as each other. Like most of the studies included in this review, they used Wits appraisal, overjet and overbite, but they also included Co-A, Co-Gn and anteroposterior molar relation. The present meta-analysis examined the five variables employed by most of the studies included (Wits, overjet, ANB, SNA and SNB).

The results confirm that skeletal anchorage is an effective treatment for improving skeletal Class III malocclusion since the five variables analysed (Wits, overjet, ANB, SNA and SNB) exhibited significant changes. When compared with other more traditional treatments such as disjunction and face mask, however, there are no differences in overjet, ANB or SNB. Wits and SNA did show a slight improvement, although the heterogeneity of the two meta-analyses was high and the number of studies analysed was low. This would indicate that there is no clear evidence that skeletal anchorage gives better results than traditional treatments. However, the systematic reviews by Feng et al. [28] and Jamilian et al. [29] found that maxillary protraction with skeletal anchorage has a greater maxillary advancement effect and seems to reduce some side effects such as mandibular rotation, extrusion of the lower molars or proclination of the upper incisors. The lower incidence of adverse effects is found again in the systematic review by Sahin et al. [30], who also concluded that skeletal effects are achieved more rapidly with this type of anchorage.

The main limitations of the present study that should be mentioned are the small number of articles included and the fact that many of the variables analysed were only recorded in two of them. Another aspect to be borne in mind is that successful treatment depends to a great extent on patient collaboration. Also, it has not proved possible to create subgroups that could have been of interest, such as differentiating between skeletal anchorage with a facemask and skeletal anchorage using intermaxillary elastic bands. In an attempt to control for publication bias, the search was conducted in four databases and was complemented by a grey literature search and hand-searching. A study that has not been included or new studies that might be conducted could alter these results.

Skeletal anchorage is an effective treatment for improving skeletal Class III malocclusion, but when compared with other traditional treatments such as disjunction and face mask there is no clear evidence that skeletal anchorage gives better results.

## Supporting information

S1 Table PRISMA 2009 checklist. (PDF)
